# Bacterial community composition in the gut content of *Lampetra japonica* revealed by 16S rRNA gene pyrosequencing

**DOI:** 10.1371/journal.pone.0188919

**Published:** 2017-12-05

**Authors:** Yu Zuo, Wenfang Xie, Yue Pang, Tiesong Li, Qingwei Li, Yingying Li

**Affiliations:** 1 School of Life Science, Liaoning Normal University, Dalian, China; 2 Lamprey Research Center, Liaoning Normal University, Dalian, China; Kliniken der Stadt Köln gGmbH, GERMANY

## Abstract

The composition of the bacterial communities in the hindgut contents of *Lampetrs japonica* was surveyed by Illumina MiSeq of the 16S rRNA gene. An average of 32385 optimized reads was obtained from three samples. The rarefaction curve based on the operational taxonomic units tended to approach the asymptote. The rank abundance curve representing the species richness and evenness was calculated. The composition of microbe in six classification levels was also analyzed. Top 20 members in genera level were displayed as the classification tree. The abundance of microorganisms in different individuals was displayed as the pie charts at the branch nodes in the classification tree. The differences of top 50 genera in abundance between individuals of lamprey are displayed as a heatmap. The pairwise comparison of bacterial taxa abundance revealed that there are no significant differences of gut microbiota between three individuals of lamprey at a given rarefied depth. Also, the gut microbiota derived from *L*. *japonica* displays little similarity with other aquatic organism of Vertebrata after UPGMA analysis. The metabolic function of the bacterial communities was predicted through KEGG analysis. This study represents the first analysis of the bacterial community composition in the gut content of *L*. *japonica*. The investigation of the gut microbiota associated with *L*. *japonica* will broaden our understanding of this unique organism.

## Introduction

The microorganisms harboring in digestive system play important roles in nutritional, immune, and defense functions [1,2]. Identification of gut microorganisms could supply essential information for understanding interaction between the microbes and host [3]. The 16S rRNA gene pyrosequencing provides a promising approach to characterize complex bacterial communities independent of culture-based enrichment [4,5].

Lampreys belonging to *Cyclostomata*, *Petromyzoniformes*, and *Petromyzontidae*, are one of the most ancient vertebrates in the world [6]. As the “live fossil”, lamprey was considered to be an excellent model for studying vertebrate evolution, embryo development, and the origin of adaptive immunity [7,8]. There were three species of lamprey including *Lampetra reissneri*, *Lampetra morii*, and *Lampetrs japonica* distributing in China north area. The morphological characteristic of lamprey is original. Their tissues and organs represent the initial stage of vertebrate evolution and development. The life cycle of lamprey is peculiar. The juvenile period of lamprey is very long at a minimum of 5 years. After metamorphosis, ammocoetes develop into adult period which can last for 1 to 4 years. The adult lampreys migrate into sea and feed on fish. During the spawning season, adult lampreys migrate to shallow water streams and die after spawning. The eggs develop and move into the next life cycle [9]. It is difficult to breed lamprey artificially under laboratory conditions on account of its peculiar life cycle. Unlike jawed vertebrates, Lampreys use variable lymphocyte receptors (VLR) consisting of leucine-rich repeats (LRR) for antigen recognition [10]. Three VLR genes including VLRA, VLRB, and VLRC have been identified in lampreys [11,12]. Still, mechanism on development of the adaptive immune system remains unclear.

This study analyzed the bacterial community structure in the hindgut contents of *L*. *japonica* by Illumina MiSeq of the 16S rRNA gene. The bacterial composition, characteristics, and potential function prediction analysis were investigated. The analysis of the bacterial community structure in lamprey intestine could not only broaden our understanding on this peculiar ancient animal but also give us clues for investigating the roles of the gut derived bacteria played in the adaptive immune functions of lampreys.

## Materials and methods

### Animal collection

This study was carried out in strict accordance with the recommendations in the Guide of the Animal Welfare and Research Ethics Committee of the Institute of Dalian Medical University. The protocol was approved by the Committee on the Ethics of Animal Experiments of the Dalian Medical University (Permit Number: SYXK2004-0029). We performed the animal experiments at the Institute of Dalian Medical University. All the handing of lampreys and all experimental procedures were made to minimize suffering. Wild Japanese lampreys (*Lampetrs japonica*) were obtained from the Tongjiang Valley, a branch of the Songhua River in Heilongjiang Province of China. The Tongjiang Valley locates in Tongjiang city (east longitude: 132°18’32”-134°7’15”; north latitude: 47°25’47”-48°17’20”), northeast of Heilongjiang Province of China. We confirm that no specific permissions were required for the activities in this river because it was not the protected or private area of river. We also confirm that the field studies did not involve endangered or protected species.

30 of male lamprey at the age of 5–6 years with size of 25 cm around were selected out, kept in Fiber Reinforced Plastic (FRP) tanks with fresh water at 4–10°C and starved for a week to remove the passing flora before dissecting. Lampreys were narcotized using ethyl 3-aminobenzoate methanesulfonate at a final concentration of 50 mg/L. After surface sterilization, three individuals of healthy ones were selected out. Gut was dissected out aseptically after collecting blood from tail-severed lampreys. Approximately 0.5 g of hindgut contents of each selected individual was gathered in sterile freezing tubes and stored at -80°C until DNA extraction.

### DNA extraction and PCR amplification

DNA was extracted from the gut contents using the E.Z.N.A Soil DNA Kit (Omega Bio-Tek, Norcross, GA, USA) according to the manufacturer’s protocol. The universal primers 338F (5’-ACTCCTACGGGAGGCAGCA-3’) and 806R (5’-GGACTACHVGGGTWTCTAAT-3’) were used for amplification of the V3-V4 region of the gut bacterial 16S rDNA gene. The 25 μL reaction mixture contained 5 × reaction buffer 5 μL, 5 × GC buffer 5 μL, dNTP (2.5 mM) 2 μL, forward primer (10 μM) 1 μL, reverse primer (10 μM) 1 μL, DNA template 2 μL, ddH_2_O 8.75 μL, Q5 DNA polymerase 0.25 μL. The thermocycling steps were as follows: 98°C for 2 min, 98°C for 15 sec, 55°C for 30 sec, 72°C for 30 sec, and a final extension step at 72°C for 5 min. PCR amplicons were purified using a DNA gel extraction kit (Axygen, China).

### Illumina MiSeq

The purified PCR products were quantitated using Quant-iT PicoGreen dsDNA Assay kit at Microplate reader (BioTek, FLx800). Sequencing library was constructed using TruSeq Nano DNA LT Library Prep Kit according to the following procedures: repair the end of the PCR products, ligation of the PCR products with sequence joint, fixation of PCR products in flow cells, purification of the sequence library using BECKMAN AMPure XP Beads, enrichment of sequencing library template, and selection of target sequences.

Sequencing library was tested in quality using Agilent High Sensitivity DNA Kit. After that, the sequencing library was quantitated using Quant-iT PicoGreen dsDNA Assay kit at Promega QuantiFluor fluorescence quantitative system. The library was sequenced using MiSeq Reagent Kit V3 (600 cycles) at MiSeq sequenator.

### Bioinformatics and statistical analysis

Sequences quality were checked according to the following procedures: Pyrosequencing reads with ambiguous bases and reads shorter than 150 bp were avoided, the overlap sequences between two reads should longer than 10 bp without mismatch bases, and the barcodes sequences used to identify valid sequences were assigned to each sample. After that, mismatch bases longer than 1 bp and more than eight repeated single bases were removed using QIIME software (Quantitative Insights Into Microbial Ecology, v1.8.0, http://qiime.org/) [13]. Then, the chimera sequences were removed using USEARCH (v5.2.236, http://www.drive5.com/usearch/).

Valid sequences were blasted using UCLUST against Greengenes database (Release 13.8, http://greengenes.Secondgenome.com/) [14] as the default database or the RDP (Ribosomal Database Project) database (Release 11.1, http://rdp.cme.msu.edu/) [15] as the standby database. The operational taxonomic units (OTUs) were defined at sequence divergences of 3%. Venn diagram presenting the shared or unique OTUs between different samples was made (http://en.wikipedia.org/wiki/Venn_diagram). The rarefaction curve evaluating the sufficient sequence depth was made using QIIME, and the rank abundance curve representing the numbers of abundant and rare OTUs was made (http://en.wikipedia.org/wiki/Rank_abundance_curve). The Chao1 estimator (http://scikit-bio.org/docs/latest/generated/generated/skbio.diversity.alpha.chao1.html) and the ACE estimator (http://scikit-bio.org/docs/latest/generated/generated/skbio.diversity.alpha.ace.html) were calculated. Besides, the Shannon diversity index (http://scikit-bio.org/docs/latest/generated/generated/skbio.diversity.alpha.shannon.html) representing the richness and evenness of community and Simpson index representing the community diversity (http://scikit-bio.org/docs/latest/generated/generated/skbio.diversity.alpha.simposon.html) were calculated. Bacteria structural composition in different taxonomic levels was displayed. The inter-individual variation on the microbiome between three individuals of lamprey was tested using Mothur software through Metastats (http://metastats.cbcb.umd.edu/) analysis [16]. Top20 of bacteria in genus level and top 7 of bacteria in phylum level with significant differences (P < 0.05) among three individuals of lamprey were displayed. The Unweighted and weighted pair-group method with arithmetic means (UPGMA) tree using beta-diversity measurements were constructed to compare the similarity of gut microbiota from *L*. *japonica* in relation to those of other aquatic vertebrates. Using GraPhlAn, dominant bacterial community could be discovered quickly [17]. Heatmaps were generated with the R-package gplots to display the composition and the abundance of OTUs quickly and flexibly. MEGAN (http://ab.inf.uni-tuebingen.de/software/megan6/) was used to construct the classification tree in order to present the composition of all the microorganisms at different classification levels [18].

The community composition in different classification levels were clustered according to the abundance distribution of the taxon units or the similarity between different samples. The classification units and samples were then ranked according to the clustering results, and were presented as the heat map. The taxon units can be distinguished between high abundance and low abundance. PICRUSt (http://huttenhower.sph.harvard.edu/galaxy/tool_runner?toll_id=PICRUSt_normalize) was used to predict bacterial function. Numbers of shared functional clusters are displayed as Venn diagram after calculating by R software according to the abundance distribution of the predicted function groups in different samples.

### Nucleotide sequence accession number

The deep sequence data have been deposited in NCBI Sequence Read Archive under accession number SRP120900.

## Results

### Richness and diversity

**Fig 1:**
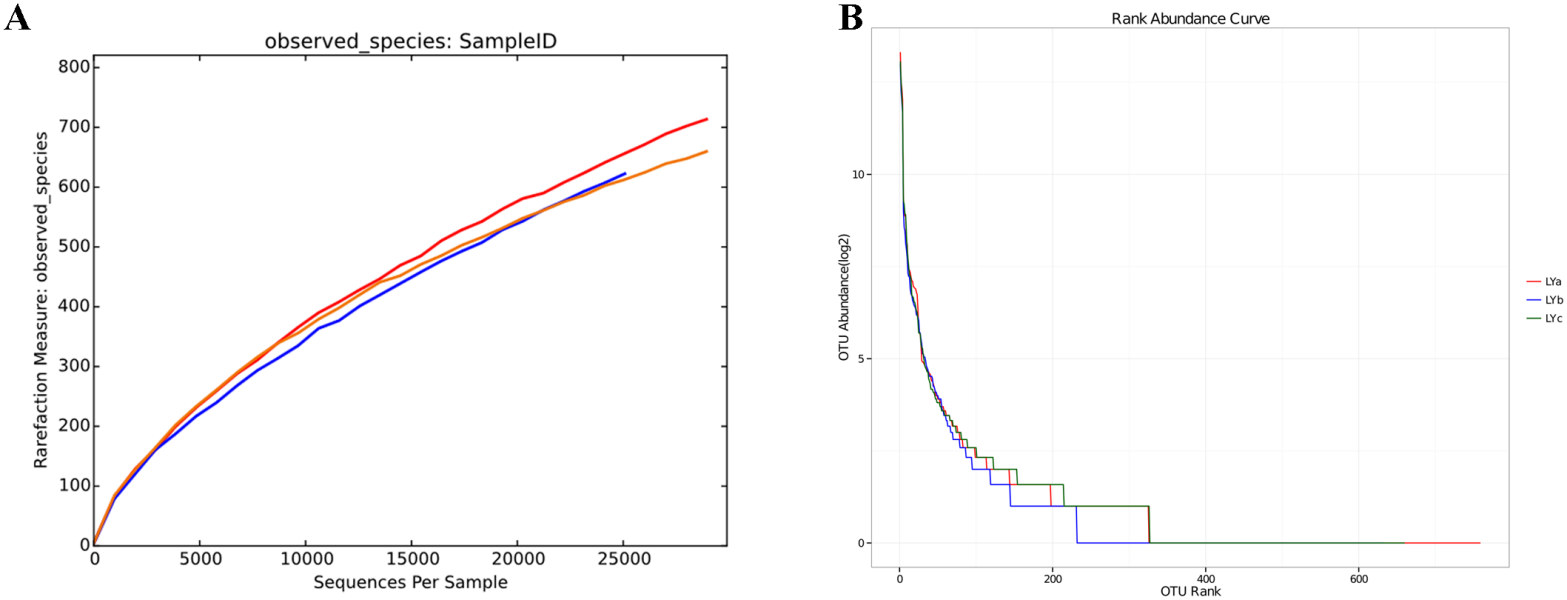
The rarefaction and the rank abundance analysis of the microbe species from hindgut contents of *L*. *japonica*. (A) the rarefaction curve; (B) the rank abundance curve.

The optimized reads ranging from 27126–33088 were obtained from hindgut contents of three individual lampreys. All of the optimized reads were classified into OTUs under different taxonomic levels (Table 1). The rarefaction curves based on the OTUs of the bacterial community in lamprey intestine reached saturation plateau, indicating that the sequencing depth were sufficient to represent the majority of microbe species (Fig 1A). The rank abundance curve representing richness of bacterial community was showed in Fig 1B. Results of the microbial diversity index including Chao1, ACE, Simpson, and Shannon were listed in Table 2.

**Table 1:** Numbers of OTUs of intestinal microbe from hindgut contents of three individuals of *L*. *japonica* under different taxonomic level.

Sample	Phylum	Class	Order	Family	Genus	Species	Unclassified
LYa	752	748	727	618	430	136	7
LYb	628	626	615	554	398	113	5
LYc	647	646	632	556	391	103	13

**Table 2:** The microbial diversity index including Chao1, ACE, Simpson, and Shannon of 16S rRNA sequence library from three individuals of *L*. *japonica* at a given rarefied depth.

Sample	Simpson	Chao1	ACE	Shannon
LYa	0.817116	325.00	502.15	3.41
LYb	0.815291	231.00	347.39	3.37
LYc	0.826510	326.00	441.42	3.51

### Bacterial community structure in the gut of *L*. *japonica*

**Fig 2:**
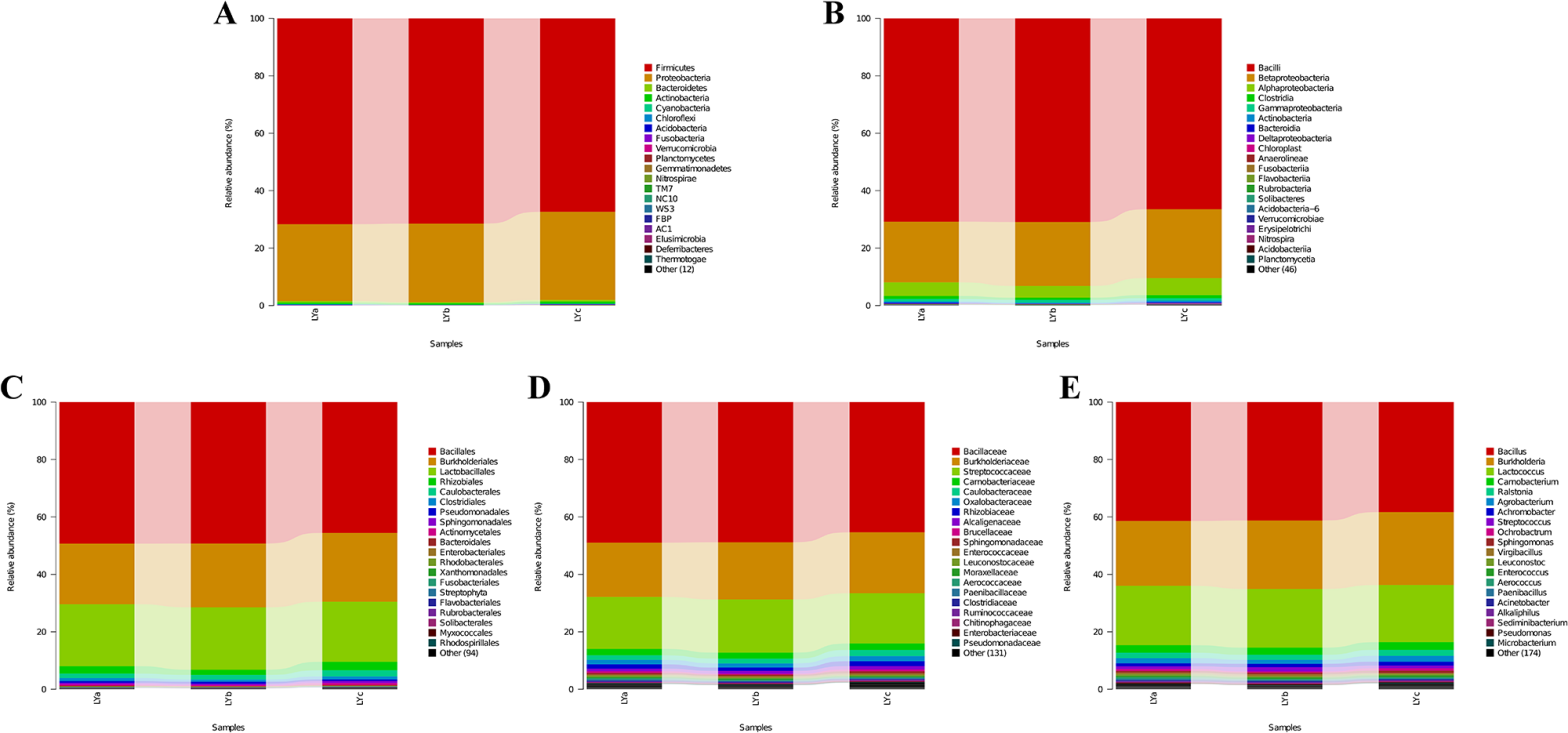
Microflora distributions of the microbe species from hindgut contents of *L*. *japonica* in five classification levels. (A) In the phylum level; (B) In the class level; (C) In the order level; (D) In the family level; (E) In the genera level.

**Fig 3:**
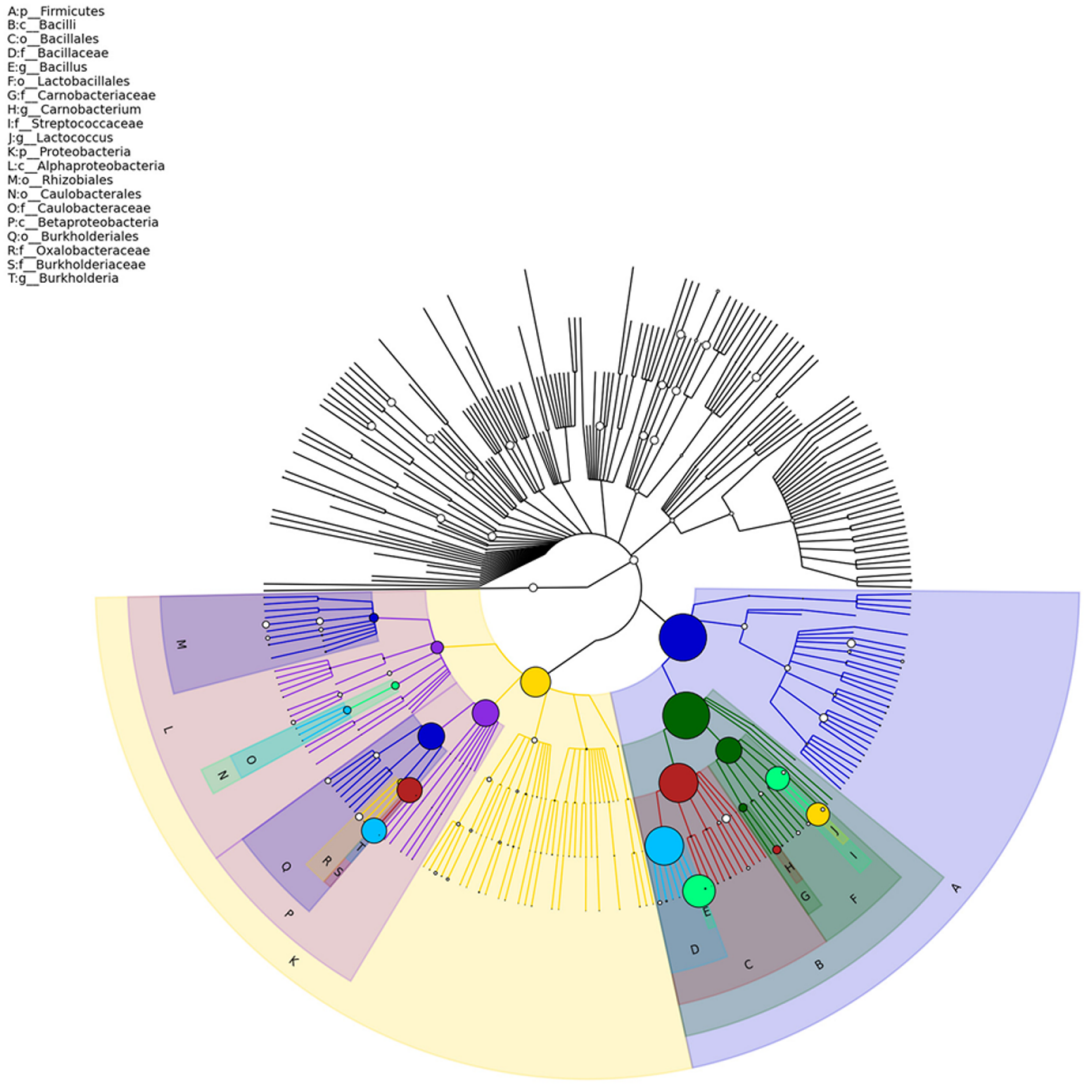
Top 20 dominant microbes in different classification level. The colored circles imply the top 20 dominant microbes in relative abundance in different classification level. The larger the radius of the circles is, the higher the species in abundance. The non-colored part implies microbes that are not in the top 20 dominant. P: Phylum; C: Class; O: Order; F: Family; G: Genera.

The composition of microbe in five classification level was analyzed (Fig 2). At the phylum level, Firmicutes (70.0%) and Proteobacteria (28.2%) are dominant members. Bacilli and Betaproteobacteria are two dominant classes, which account for 69.1% and 22.4%, respectively. At the order level, Bacillales, Burkholderiales, and Lactobacillales are three dominant members, which account for 47.8%, 22.3%, and 21.3%, respectively. Bacillaceae (47.0%), Burkholderiaceae (19.7%), and Streptococcaceae (17.8%) are dominant members at the family level. At the genera level, *Bacillus*, *Burkholderia*, and *Lactococcus* are detected as dominant members, which accounted for 33.2%, 19.7%, and 16.7%, respectively. Using GraPhlAnl, top 20 dominant members at different classification level are displayed as the classification tree (Fig 3). The abundance of microorganisms in different samples is displayed as the pie charts at the branch nodes in the classification tree (S1 Fig).

### Comparison of the bacterial communities in the gut of different individuals of *L*. *japonica*

**Fig 4:**
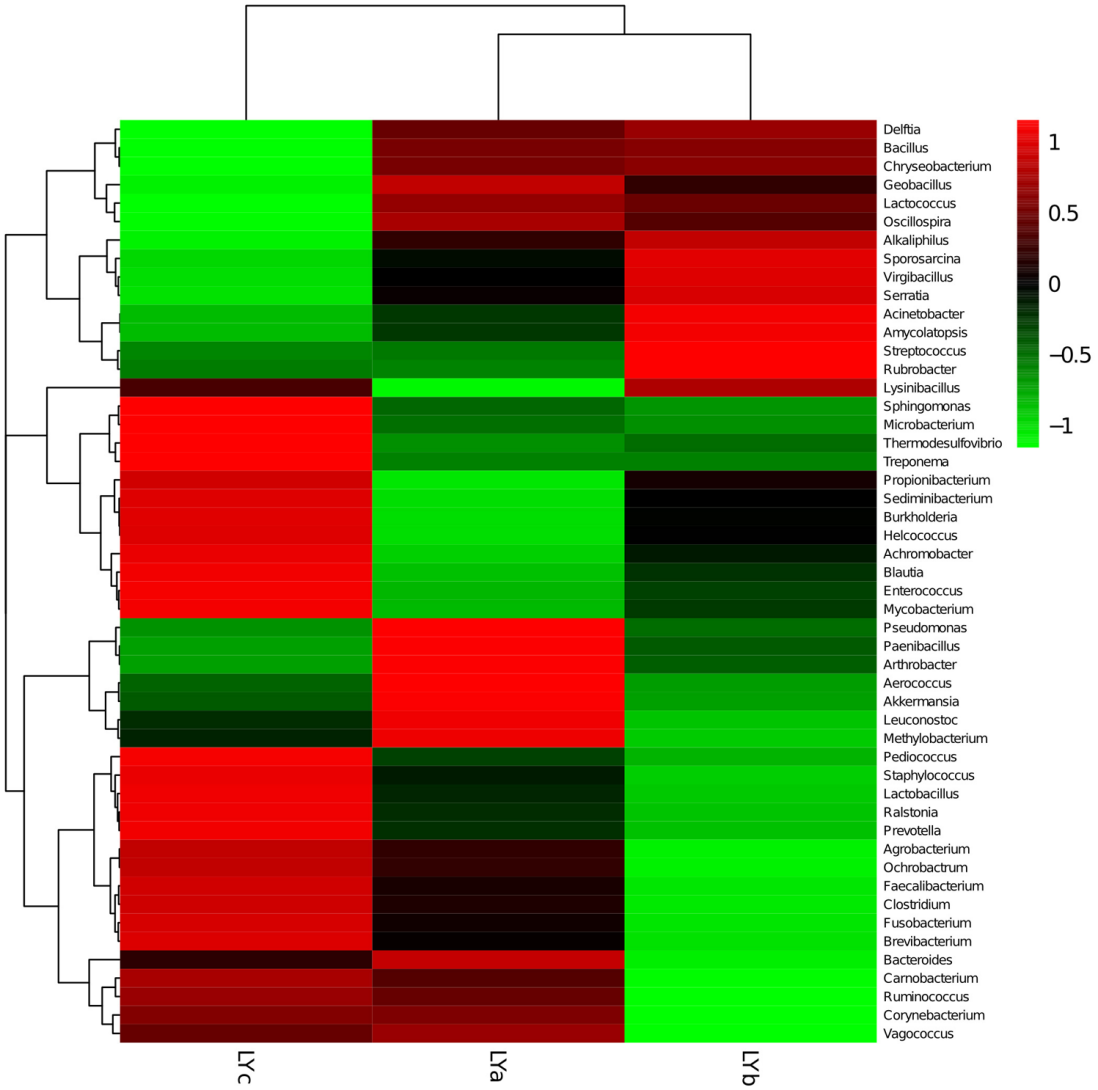
Hierarchically clustered heatmap analysis of the top 50 genera in intestine of different individuals of lamprey. The relative values for bacterial genera are indicated by color intensity with the legend indicated at the top right corner of the figure.

**Fig 5:**
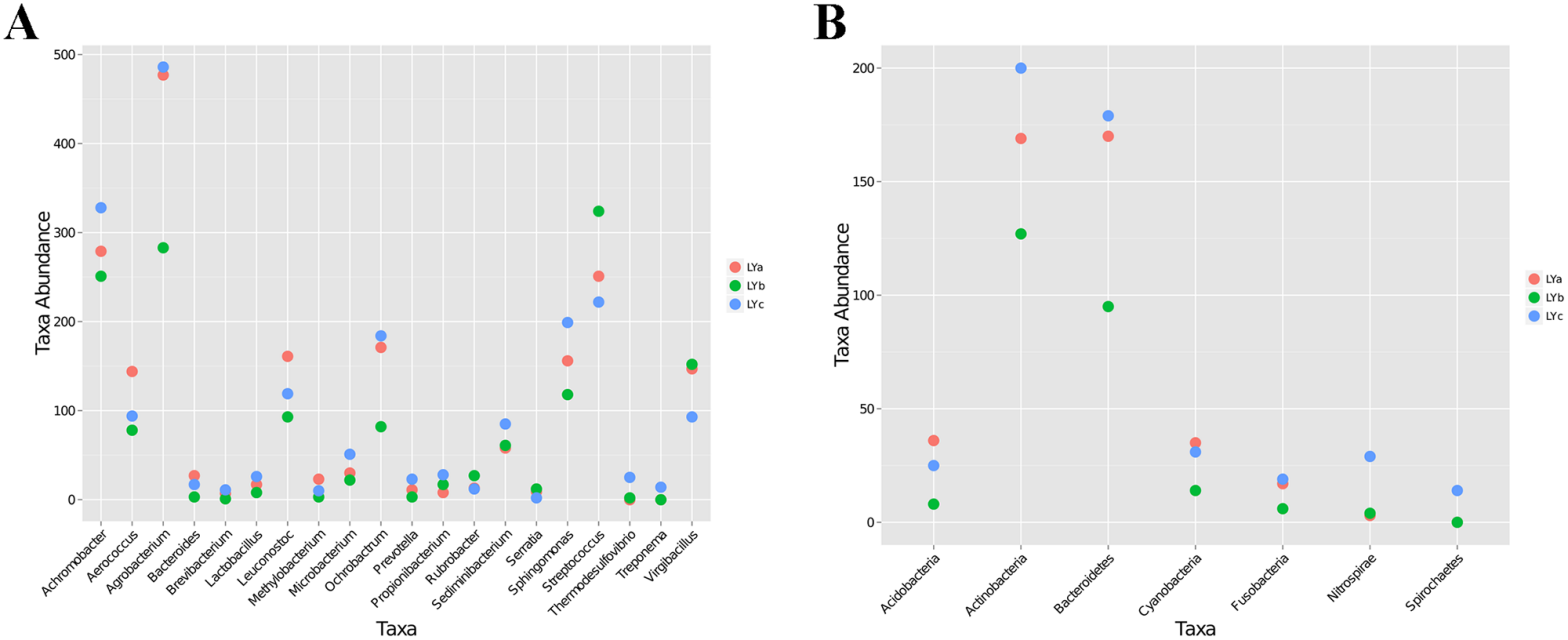
The pairwise comparison of bacterial taxa abundance in intestine between three individuals of lamprey. (A) top 20 member of bacteria in genera level; (B) top 7 member of bacteria in the phylum level.

**Fig 6:**
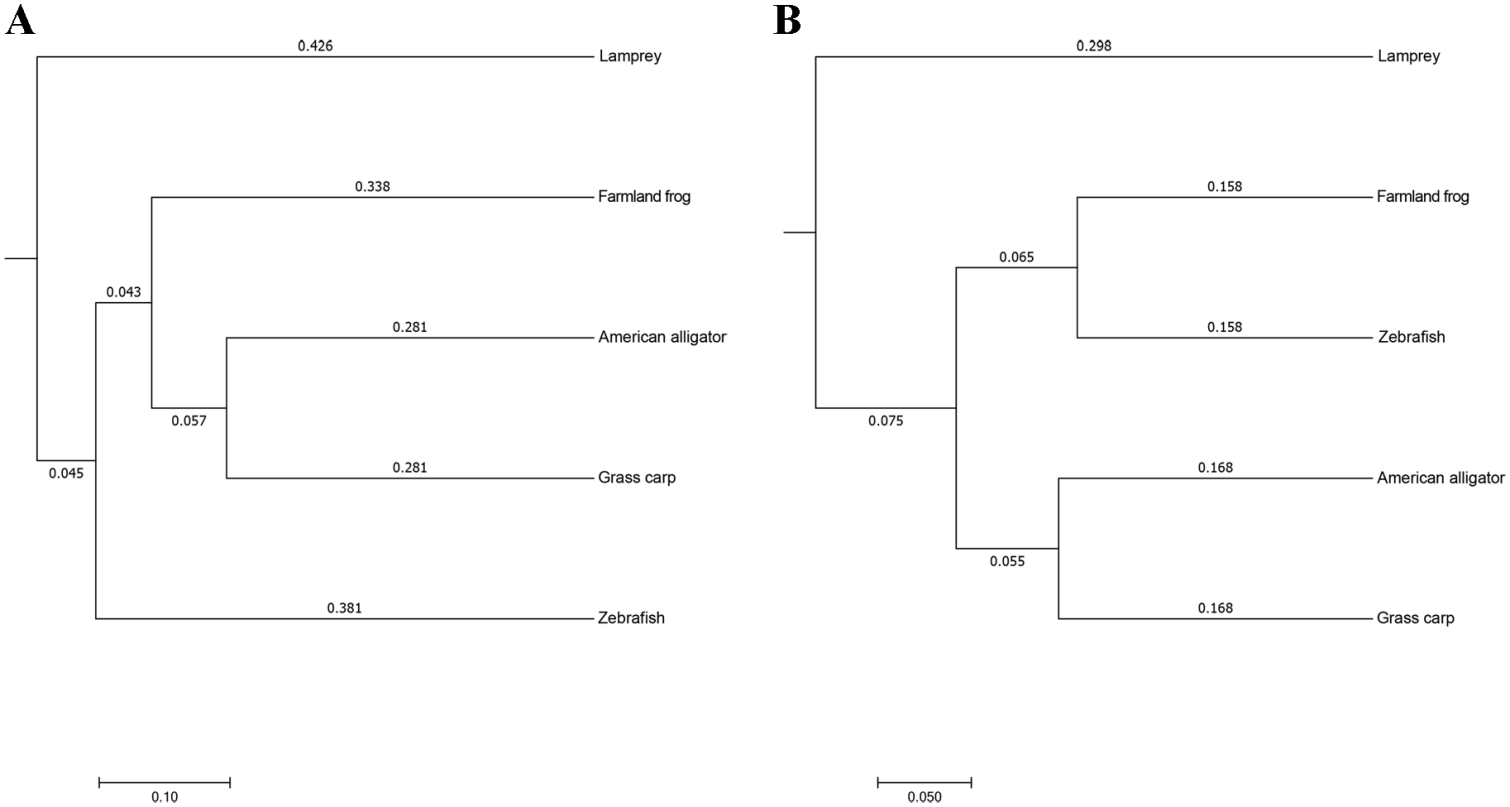
UPGMA analysis clustering analysis based on UniFrac distance matrix. (A) UPGMA tree based on unweighted unifrac distance matrix; (B) UPGMA tree based on weighted unifrac distance matrix. The UPGMA analysis revealed that gut microbiota derived from *L*. *japonica* display little similarity with other aquatic organism of Vertebrata. Lamprey: gut microbiota deep sequence data derived from *L*. *japonica* in this study; farmland frog, American alligator, grass carp, and zebrafish: gut microbiota deep sequence data derived from public database.

**Fig 7:**
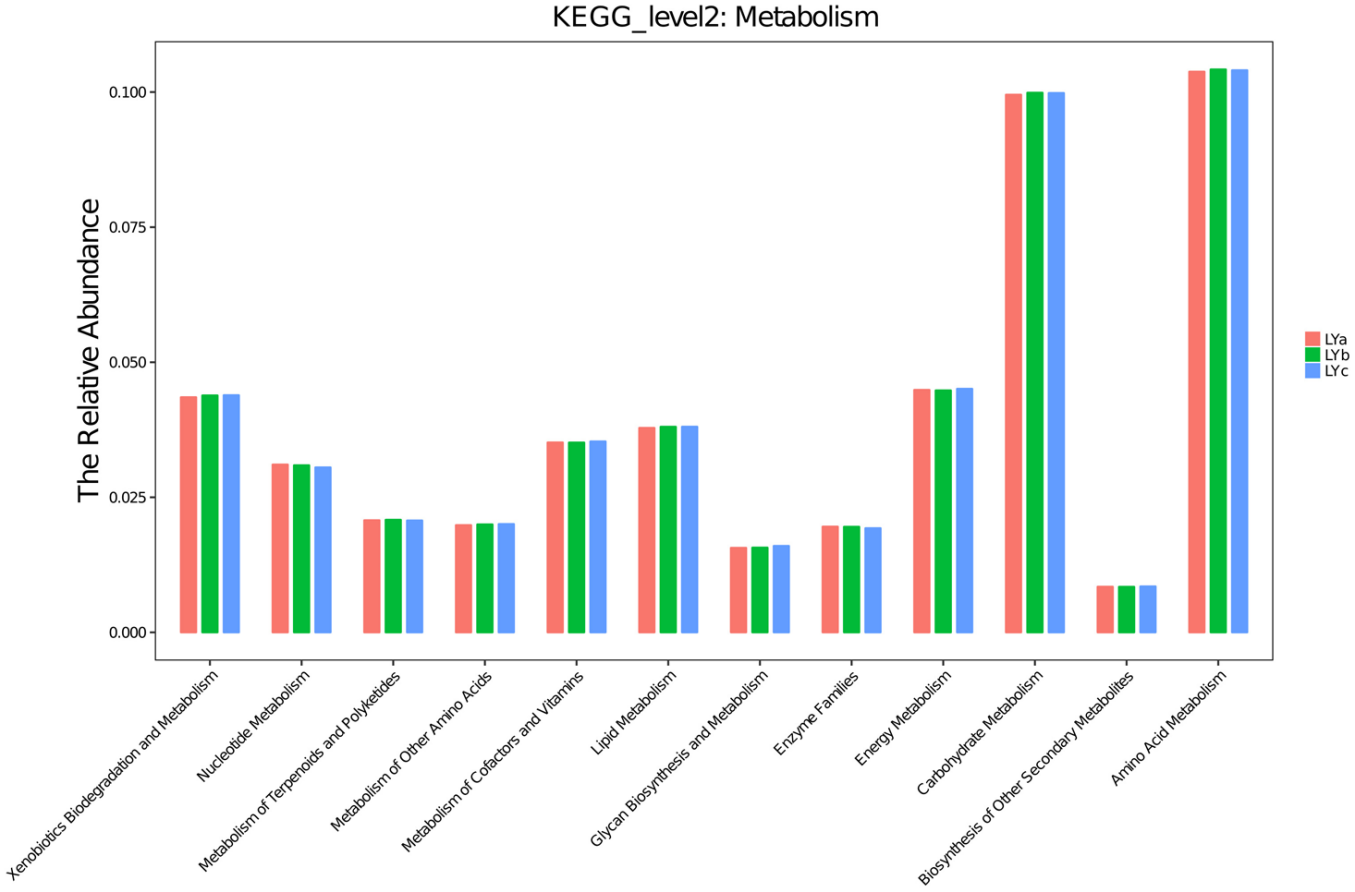
The relative abundance of intestinal microbe involving in the subfunction of metabolic pathways predicted through KEGG pathway database.

**Fig 8:**
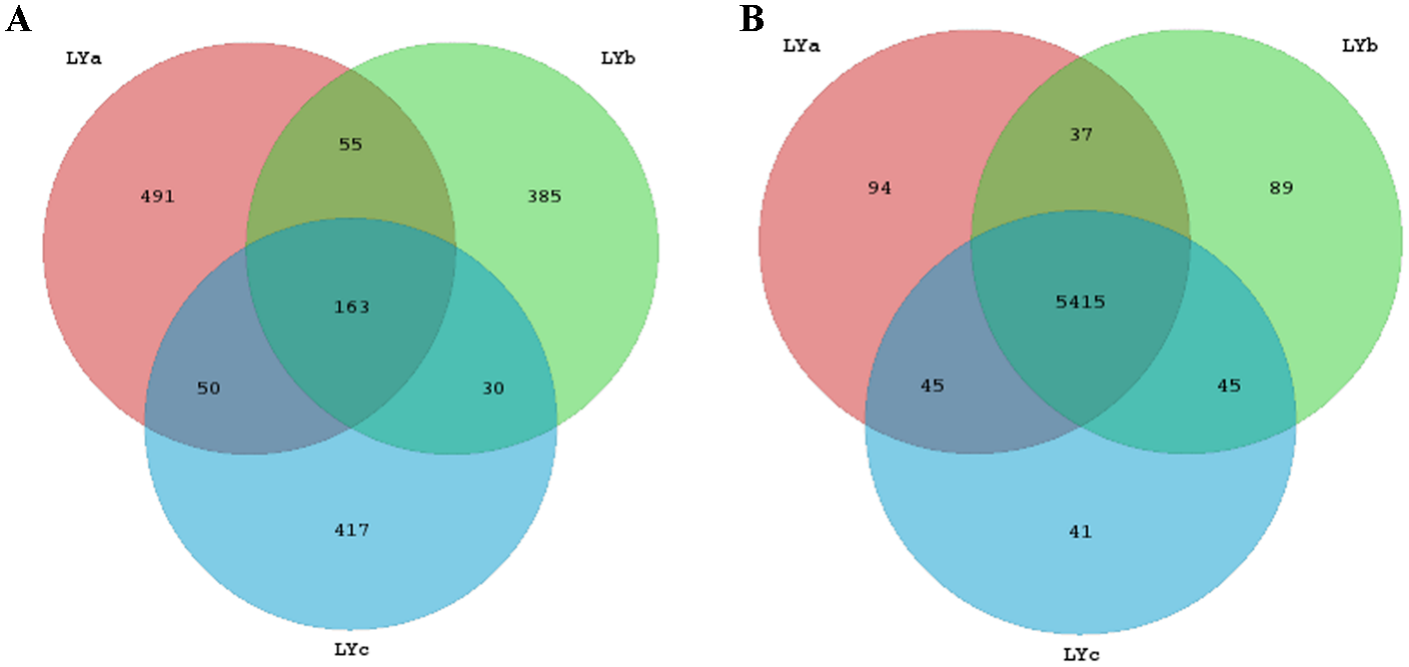
Venn diagram showing shared OTUs and shared functional clusters of microbes in different individuals of lamprey. (A) Shared OTUs; (B) Shared functional clusters of microbes.

A heatmap representing the differences between individuals of lamprey were displayed in Fig 4. After data correction, the top 50 genera with different abundance can be found according to the color variations. Red represents the genus in high abundance, while green represents low abundance. The inter-individual variations on the microbiome between three individuals of lamprey were displayed in Fig 5. Top 20 of bacteria in genus level and top 7 of bacteria in phylum level can be detected. Results indicated that all of the top 20 of bacteria in genus level and top 7 of bacteria in phylum level with differences in relative abundance are non-dominant members. Therefore, there are no significant differences on gut bacteria abundance between individuals of lamprey. The similarity of gut microbiota from *L*. *japonica* in relation to those of other aquatic vertebrates was compared. An analysis of current data shows that gut microbiome composition of *L*. *japonica* was distinct (Fig 6). Therefore, they did not show phylosymbiosis of *L*. *japonica* with other aquatic organism of Vertebrata. The relative abundance of microbe is summarized according to the functional clusters predicted through KEGG pathway database (Fig 7). Besides, the shared OTUs between different individuals of lamprey are displayed in Fig 8A. There are 163 OTUs existing in all of the three samples, while 491, 385, and 417 OTUs are distributing in each group of lamprey. Also, the shared functional clusters of microbes in different individuals of lamprey are displayed in Fig 8B. There are 5415 shared function clusters existing in all of the three samples, while 94, 89, and 41 functional clusters of microbes distributing in each group of lamprey.

## Discussion

In the present study, gut content library from *L*. *japonica* contained 32 different phyla, the number of which is much higher compared with gut microbiota from *L*. *morii* [19]. The difference of gut microbiota between the two species is probably due to their different life cycle. Unlike *L*. *japonica*, *L*. *morii* live in fresh water throughout their life cycle. *L*. *japonica*, however, migrates into sea and feed on fish after metamorphosis. Therefore, the gut microbiota in *L*. *japonica* is more complex than in *L*. *morii*.

Bacteroidetes and Proteobacteria were detected as the dominant phyla in the intestine of *L*. *morii* [19]. In *L*. *japonica*, however, Firmicutes and Proteobacteria are dominant members. Proteobacteria are a major phylum of gram-negative bacteria, which are also dominant in the intestine of other species of aquatic organisms, such as *Ctenopharyngodon idellus*, *Megalobrama amblycephala*, *Carassius auratus*, and *Hypophthalmichthys nobilis*. In the intestinal contents and mucus of *Oncorhynchus mykiss*, most of the phylotypes were affiliated with Proteobacteria (>70% of the total) [20]. Firmicutes, another dominant phylum in *L*. *japonica* intestine, was not detected in *L*. *morii*. However, Firmicutes accounted for 18.63% in the *H*. *nobilis* intestine. At the class level, the bacterial community consisted mainly of Betaproteobacteria in the digestive tract of the aquatic crustacean *Daphnia magna* [21]. Some species of *Bacillus* sp. such as *Bacillus pumilus* SE5, was proven as the probiotics which could shape the intestinal microbiota and mucosal immunity in grouper *Epinephelus coioides* [22]. At genera level, *Bacillus* sp. was detected as dominant member in *L*. *japonica* intestine. The roles that *Bacillus* sp. play in the development of adaptive immune system deserves further study. *Lactococcus*, a kind of lactic acid bacteria (LAB), possess a major potential in biopreservation strategies [23]. LAB are commonly associated with the mucosal surfaces of the gastrointestinal, which can enhance host protection against pathogens [24]. LAB can produce one or more antimicrobial active metabolites and antimicrobial peptides. Their antagonistic and inhibitory properties make them to be highly valuable species for fisheries and aquaculture. *Lactococcus* are also dominant genera in *L*. *japonica* intestine. The selection and functional analysis of *Lactococcus* species from intestinal microbiota in *L*. *japonica* will be needed. *Aeromonas*, a widely distributed bacterium in aquatic environments and the gut microbiota of other fish [20,25], have been commonly used as probiotics [26]. As a dominant member in intestine of *L*. *morii*, *Aeromonas* was speculated to be involved in the lamprey immune response. However, *Aeromonas* was not detected in *L*. *japonica* intestine. Studies revealed that single or mixed group of microbiota in host intestine could induce the accumulation of specific populations of immune cells in gnotobiotes [27,28,29]. The characterization of key microbiota essential for adaptive immune response of *L*. *japonica* and the identification of novel genes related with lamprey immune response will be further studied.

The microbiome compositions do not reflect host phylogenetic affiliations for all Vertebrata. Adaptive immunity of jawless vertebrates and jawed vertebrates has evolved two different molecular strategies for antigen recognition. Bony fish, amphibians and reptiles, which belong to jawed vertebrates, display more and more distant vertebrate phylogeny with lamprey [30]. Also, the gut microbiota of *L*. *japonica* in relation to those of grass carp (bony fish) [25], frog (amphibians) [31], and alligator (reptiles) [32] were different. Although Proteobacteria and Firmicutes were dominant in the intestinal mucosa of grass carp, *Aeromonas* spp. and *Shewanella* spp. comprised the dominant cultured bacteria at the genus level [25]. Frogs, which belong to amphibians, display changes in dominant intestinal microbes with different diets [31]. The dominant microbial phylum Bacteroidetes can be detected in frogs inhabiting natural habitats. However, Bacteroidetes in natural environments was replaced by the microbial phylum Firmicutes in farmland frogs. At genus level, the dominant members are *Treponema*, *Roseomonas*, and *Clostridium* in frogs inhabiting farmland habitats. In frogs inhabiting natural habitats, however, the *Bacteroides* and *Rikenella* are dominant members at genus level. In American alligator, the gut microbiome comprised of Fusobacteria, but depleted in Bacteroidetes and Proteobacteria [32]. This composition in gut bacteria is unique from other vertebrate gut microbiomes. In conclusion, the gut microbiota of *L*. *japonica* displays little similarity with other aquatic organism of Vertebrata because of their different diets and living environments.

Given the importance of microbiota in the development and maintenance of the immune system of host, the characterization of the bacterial community in intestine of *L*. *japonica* will give clues for understanding the unique immune systems of lamprey. The analysis of the composition of intestinal microbiota will provide strategy for artificial breeding of *L*. *japonica*.

## Supporting information

S1 Fig The classification tree displaying the phylogenetic relationship and the abundance of microorganisms. The pie charts in the branch nodes displaying the abundance of microorganisms in different samples. The larger the sectorial area, the higher the abundance.
(PDF)
