# Supplementary figures and images for: Bacterial community composition in the gut content of Lampetra japonica revealed by 16S rRNA gene pyrosequencing
Source: PLoS One. 2017 Dec 5;12(12):e0188919. doi: 10.1371/journal.pone.0188919 (PMC5716533; doi:10.1371/journal.pone.0188919)

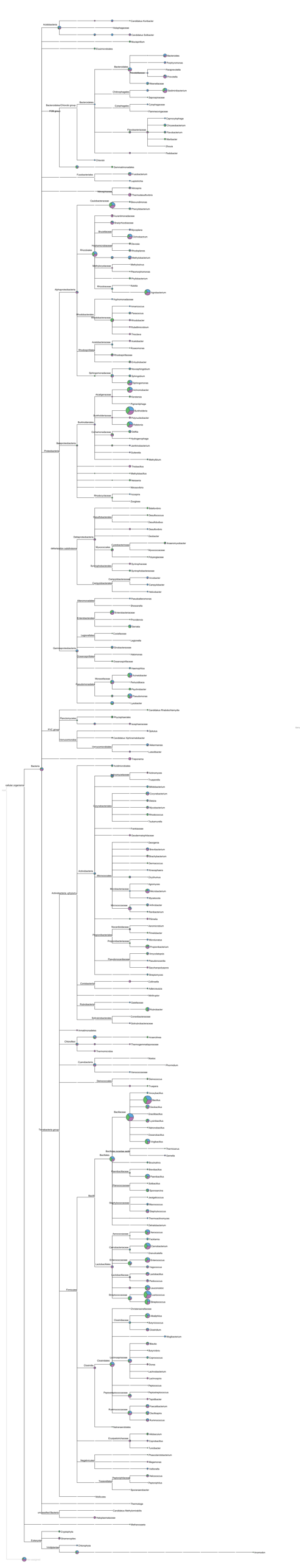

Legend:

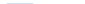 LYa   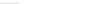 LYb   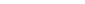 LYc

Supplement: S1 Fig — The pie charts in the branch nodes displaying the abundance of microorganisms in different samples. The larger the sectorial area, the higher the abundance. (PDF) [file pone.0188919.s001.pdf]
